# Pioneering and sustaining capacity building in diabetes care: journey of the CCEBDM program

**DOI:** 10.1186/s12909-026-08896-3

**Published:** 2026-02-27

**Authors:** Pushkar Kumar, Swastika Chakravorty, Santosh Kumar, Deepak Monga, Dilip Jha, Rajesh Kumar Mishra, Ranjit Mohan Anjana, Viswanathan Mohan, Ranjit Unnikrishnan

**Affiliations:** 1 Training Division Public Health Foundation of India, New Delhi, India; 2 Dr. Mohan’s Diabetes Education Academy, Chennai, India

**Keywords:** Diabetes, Primary physicians, Continuing medical education (CME), Non-communicable diseases (NCDs), India

## Abstract

**Background:**

Given the rising burden of diabetes and related complications in India, primary care physicians have been identified as crucial to mitigate the increasing burden at the population level. Therefore, effective training programs tailored to enhance their competencies in diabetes care become indispensable for improving patient health outcomes.

Catering to this need, the Certificate Course on Evidence-Based Diabetes Management (CCEBDM) offers a structure that equips frontline care health providers with the necessary skills to provide evidence-based diabetes care.

**Methods:**

The paper mostly presents descriptive and qualitative insights to explain the various components of the CCEBDM program that have contributed to its sustained success and impact.

**Results:**

To date, a total of 17,557 primary care physicians (PCPs) have enrolled for the training in the CCEBDM program. The participants comprise a diverse group of physicians, encompassing clinical experience ranging from 3 to 54 years (mean: 10.4 years). Respondents have reported measurable improvements in both clinical practice and patient care following CCEBDM training. Participants from a small narrative program evaluation based on retrospective survey data reported an increase in the average patients per month from 80 to 140, showing a 76% increase in the number of patients managed. Given the extensive number of PCPs trained by CCEBDM, this indicates a significant impact in forwarding evidence-based management of diabetes in India.

The undebatable decade-long success of the CCEBDM program may be attributed to two main factors. The first factor consists of adaptability & innovative methods that have helped evolve the program in tune with the changing epidemiological needs and gaps in diabetes management in India. The second factor consists of robust and valid features that have ensured and preserved the quality of the program.

**Conclusions:**

The paper outlines a forward-looking agenda for optimizing the program’s impact on transforming India’s diabetes care landscape. Given the rising incidence of non-communicable diseases and the acute shortage of specialized care in underserved areas, the paper explains why programs like CCEBDM are well-positioned to serve as a strategic model for decentralized capacity-building.

**Supplementary Information:**

The online version contains supplementary material available at 10.1186/s12909-026-08896-3.

## Background

Diabetes mellitus affects millions of individuals and families worldwide, touching lives across every region and community. As one of the leading causes of death, its impact is both personal and far-reaching [1]. The number of people living with diabetes is projected to rise sharply—from 589 million in 2024 to 853 million by 2050 [2]. South Asia alone accounts for over 22% of global cases [3], with prevalence rates varying widely, from 4% in Nepal to 23% in Sri Lanka [4]. The percentage of patients achieving combined ABC (HbA1c, blood pressure control, and LDL cholesterol) control in India, although improving, remains at 7% currently [5]. For every individual affected, timely diagnosis and careful management of blood glucose can make a profound difference, helping to prevent or delay the complications that disrupt lives [6].

Diabetes mellitus is not just a statistic in India—it is a lived reality for over 101 million people and their families, as reported by the ICMR – INDIAB Study [7, 8]. By 2040, this number is expected to reach 123 million, meaning nearly one in five people with diabetes worldwide will be in India [1, 4]. The incidence of diabetes, that is, the new onset of diabetes, is also large [9], and an unhealthy diet [10] along with physical inactivity [11] are the main drivers of the diabetes epidemic. If it is not controlled, it can lead to complications [12–14]. Another challenge is that in Indians, it occurs at a young age [15] which increases the risk of complications. Behind these numbers are individuals navigating daily challenges, from managing their blood sugar to seeking guidance on lifestyle changes. The burden on India’s health system is immense, especially at the primary care level, where most patients first seek help. Relying solely on specialist care is not feasible, given the scale of the problem. Instead, primary healthcare professionals—often the first point of contact—become crucial partners in each patient’s journey. Ongoing education and clinical upskilling of these professionals are essential for addressing this chronic, complex disease and for ensuring that every patient has a chance to achieve better health outcomes and an improved quality of life [16–18].

### Role of Primary Care Physicians (PCPs) in diabetes management

The limited availability of specialists and their inequitable distribution over regions and locations exacerbates the challenge of managing the huge burden of diabetes in India. Data shows that a large share of the population with diabetes in India resides in rural areas, underscoring the importance of transitioning diabetes care from specialist-centered approaches to community and primary health care systems [19]. Although India has a well-established public healthcare infrastructure providing preventive and curative services across primary, secondary, and tertiary levels, management of non-communicable diseases (NCDs), including diabetes, remains suboptimal [20].

The need to reinforce primary health care, especially as the first tier of health care, is a critical step in resolving this issue. This is especially relevant in the context of India’s dismal doctor population ratio of 1:1800 [21] and the chronic maldistribution of specialists, the bulk of whom practice in metropolitan areas. Additionally, the fact that two out of every three diabetes cases are identified at the first point of contact by non-specialist healthcare providers suggests that they are the primary point of contact for these services. Enhancing the capacity of PCPs through targeted training and resources is crucial for improving access to effective diabetes care, particularly in underserved and rural areas. By empowering primary healthcare systems, India can build a more equitable and sustainable framework to combat the rising burden of diabetes and its associated complications.

Most often, family doctors act as the first contact between people and the healthcare system as they are easily accessible, acceptable, and affordable. Their closeness to the population and the fact that they are the first contact point in the diagnosis, management, and prevention of illnesses provide them a unique and strategic angle to ease the burden that comes with caring for people suffering from diabetes. There is sufficient evidence across countries to assert that patients who have regular access to PCPs often incur less cost and have better health outcomes than those who do not [17, 22]. Effective training programs tailored to enhance their competencies in diabetes care are, therefore, indispensable for improving health outcomes at the population level.

To address the emerging need for a swift and accurate response to manage diabetes, the Certificate Course on Evidence-Based Diabetes Management (CCEBDM) was launched by Dr. Mohan’s Diabetes Education Academy (DMDEA) and the Public Health Foundation of India (PHFI) in 2010. This program aims to improve PCPs’ clinical understanding, diagnostic skills, and management of diabetes. The course specifically intends to equip frontline care health providers with the necessary skills to provide evidence-based diabetes care, which consists of comprehensive care to ensure early diagnosis, customized treatment, and detection and early care of complications.

Through a structured curriculum that combines theoretical learning with practical insights, the course addresses key aspects of diabetes management, such as lifestyle modification counselling, pharmacological interventions, and monitoring of glycaemic control. The initiative not only enhances the clinical capabilities of PCPs but also bridges the gap between specialist care and community-based healthcare delivery. By empowering PCPs to effectively manage diabetes within their practice settings, the CCEBDM contributes to strengthening the overall healthcare system’s capacity to tackle the escalating diabetes epidemic.

### Program background

The objective of the CCEBDM program is to enhance the knowledge, skills, and competencies of PCPs in diabetes care. The CCEBDM program was initially launched to improve diabetes care by equipping PCPs with evidence-based skills and knowledge. The program also aims to establish a network of PCPs with diabetes care specialists for improving referral and patient outcomes. In its latest form, PHFI is implementing CCEBDM as a 12-part modular program, from January 2025 to December 2025, with once-a-month sessions on weekends at 84 centres across India. The format has now evolved to a hybrid mode, with a few sessions being conducted offline while the others are being conducted online. This adaptation was done based on the feedback from the faculty and the participants in the previous batches. The continued popularity and uptake of the course is a testimony to its relevance in the current scenario of epidemiology in India.

The 2016 commentary by Bhalla et al. [23] highlighted the program’s innovative structure and its potential to bridge knowledge gaps in primary care through targeted training [23]. It elaborated that from its very genesis, the CCEBDM program was aimed at improving patient outcomes by enabling PCPs in clinical decision-making by apprising the physicians with the latest developments in the field of diabetes management and to deal with it more efficiently in their day-to-day undertakings. As the program marks its 15th anniversary, the present paper assesses its sustained reach, scalability, and pioneering approaches that have set a standard in diabetes care training.

#### Program objectives

This program aims to strengthen physicians’ capacity to deliver evidence-based care by integrating updated guidelines, clinical practices, and contextualized case management strategies.

In addition to its core objective, the program also pursues several secondary goals. It seeks to develop and periodically update a standardized teaching protocol and educational module that facilitates structured, evidence-informed learning in diabetes management. Another important aim is to build a collective network comprising PCPs and diabetes specialists, thereby encouraging peer learning, exchange of knowledge and ideas, and referral linkages from primary and preventive care to curative care. Furthermore, the course is designed to keep practitioners abreast of the latest scientific and clinical advancements in the field of diabetes, ensuring their practices remain current and responsive to emerging evidence.

#### Eligibility criteria

The program is intended for practicing medical professionals who have earned a Bachelor of Medicine and Bachelor of Surgery (MBBS) degree. Applicants must have a minimum of three years of clinical experience to be eligible for enrolment. This prerequisite ensures that participants have adequate clinical exposure and are positioned to integrate the acquired knowledge into real-world practice effectively.

#### Certification criteria

To qualify for certification under the CCEBDM program, participants must fulfil a series of academic and participation requirements. First, they are required to attend atleast ten out of the twelve monthly online instructional sessions. Theses sessions include pre- and post tests for each module to gauge knowledge acquisition and retention. In addition to this, they must also submit three interim descriptive assignments after the completion of Modules 4, 7, and 10. These assignments serve as determinative assessments to reinforce learning and application of content. 

Finally, participants must appear for and successfully pass a comprehensive exit examination administered alongside Module 12. The exam consists of 50 multiple-choice questions (MCQs), which must be completed within one hour. A minimum score of 50% is required to pass this final assessment and obtain the course certification.

## Methods

### Objective

This paper presents a descriptive narrative of the evolving history of the Certificate Course in Evidence-Based Diabetes Management (CCEBDM): its design, development, and sustained reach to enhance diabetes management in India. The analysis is focused on three thematic areas discussed below.

First, the analysis pinpoints and scrutinizes the salient features that have resulted in the program’s uninterrupted success for over a decade. These factors include modular in-text evidence-based curricula, blended learning integrating theory and clinical mentorship, strong collaborative partnerships, comprehensive monitoring and evaluation (M&E) frameworks, and a purpose-built digital program.

The second part of the paper looks at how the program adopted innovative strategies and managed to overcome the significant challenges in a rapidly changing and highly competitive ecosystem.

Finally, the paper describes the evolution, participant perception, and geographical expansion of the program in the in last decade, starting from 2010. To explore the participants’ perception of the impact of CCEBDM training, a retrospective impact survey was designed. The assessment was not designed as a formal impact evaluation; rather, it aimed to provide contextual and illustrative insights into perceived practice-level changes among a subset of programme alumni, complementing the broader narrative of the programme’s scale, evolution, and sustainability (Refer to S1). The questionnaire was designed by the research team at the Public Health Foundation of India (PHFI) in consultation with academic experts. It included components focusing on self-reported knowledge retention, change in clinical practices, and perceived professional impact of the course. The tool was based on the learning objectives and evaluation framework of the CCEBDM program.

### Ethics approval and consent to participate

The present manuscript utilizes aggregated, de-identified data from multiple cycles of the Certificate Course in Evidence-Based Diabetes Management (CCEBDM) program conducted over 15 years. All data collection activities during individual program cycles (including baseline and endline assessments) were reviewed and granted exemption from ethical approval by the institutional ethics committee, as they involved routine programmatic monitoring and evaluation without the use of identifiable personal information.

Additionally, a retrospective assessment was conducted with prior CCEBDM participants to understand the long-term perceived effects of the program. Respondents were informed about the purpose of the survey, the nature of participation, and data privacy and confidentiality before participation. The dataset used in this paper from that study is aggregated and anonymized, containing no personally identifiable information. Hence, a separate ethical approval was not sought for this secondary analysis, in accordance with institutional ethical standards. The survey items were derived from endline evaluation tools administered at the conclusion of each CCEBDM cycle, for which ethical approvals had been obtained as part of routine programme evaluation. The details of the ethics committee approval for the endline evaluation for the latest cycle of CCEBDM are as follows: *Name of the IEC: Institutional Ethics Committee (PHFI-IEC); TRC-IEC No: PHFI-IEC/2526/N/Exem/29; Date: 20/01/2026*.

## Components for sustained efficacy

### Partnerships and multi-tiered accountability framework

The long-term capacity building in diabetes management for primary care physicians throughout India has been made possible by CCEBDM’s distinctive public-private partnership model, which is a key component of its sustainability and success. Key stakeholders are included in this partnership framework, and their cooperation has made it possible for the program to continuously grow and be held accountable at all implementation levels. CCEBDM includes several stakeholders who share the goal of raising the standard of diabetes care and making it available to diabetics nationwide. The stakeholders with a common vision comprise the implementing partner, academic partner, educational funding partner, national experts, faculty, observers, and primary care physicians.

As discussed above, the program is academically supported by Dr. Mohan’s Diabetes Education Academy in Chennai, India, a leading institution in diabetes education, and implemented by the Public Health Foundation of India (PHFI). The majority of funding comes from industry partners, whose contributions guarantee the program’s financial sustainability and allow PHFI and its partners to reach a large number of participants. Participants in the program pay a moderate course fee to reaffirm their commitment and accountability. In addition to promoting program continuity, this strategy gives the participating doctors a sense of ownership, increasing the likelihood that they will finish the course and integrate the learning from the course in their clinical work [24, 25].

The involvement of a diverse set of stakeholders reinforces accountability and quality control [26]. As advisors, fifteen national experts—leading endocrinologists and diabetologists from leading institutions across the country —contribute to the meticulous creation of training materials. They play an essential role in designing the curriculum, staying current with clinical developments, and ensuring the relevance of course content. The experts are also involved in supervising training sessions, providing direct oversight and guidance to ensure high standards in delivery.

Beyond curriculum design, the training of primary care physicians (PCPs) is conducted by seasoned endocrinologists and diabetologists. This mentorship from specialists who are active and have established reputation in the field enhances the credibility of the program and ensures that participants receive expert guidance, which is integral to applying evidence-based practices in diabetes management.

Another vital component of CCEBDM’s partnership model is the involvement of over 30 public health professionals who serve as observers/facilitators during training sessions. These observers/facilitators monitor the course delivery, ensuring that it adheres to standardized practices and that each module is presented consistently across sessions. The observers/facilitators also develop session reports after each session based on their observations during the session. The program’s integrity and quality depend heavily on this multi-tiered accountability system, which includes checks from public health monitors and clinical experts. The overall details of the multitude of stakeholders involved in each cycle of CCEBDM training is provided in Table 1.

**Table 1:** Details of various stakeholders involved in each cycle of CCEBDM training

Cycle/Batch	Date of Launch	States & UTs	No. of Centres	National Experts	Faculty	Observer
						/Facilitator
Cycle-I	8th Aug-2010	18	100	15	128	61
Cycle-II	11th Dec. 2011	20	119	15	149	84
Cycle-III	24th Feb. 2013	20	134	15	164	84
Cycle-IV	15th Mar. 2015	24	130	16	162	61
Cycle-V	7th May 2017	22	137	16	162	85
Cycle-VI	12th Jan. 2020	22	137	16	162	85
Cycle-VII	9th Jan. 2022	21	103	15	122	30
Cycle-VIII	9th July 2023	21	88	15	104	30
Cycle-IX	19th Jan. 2025	20	84	12	97	30

Through this structure, CCEBDM has successfully developed a robust accountability framework that strengthens program fidelity and improves results by involving stakeholders at different levels, including academic partners, industry funders, clinical experts, trainers, and public health observers. In addition to sustaining the program’s operations, this collaborative model fosters trust among all the stakeholders, making CCEBDM a model for effective, scalable healthcare education programs in India.

### Evidence-based updated curriculum & teaching method

The CCEBDM program has adopted a structured approach to curriculum review, incorporating the latest evidence-based practices and international diabetes guidelines to maintain its relevance. This dedication to keeping the curriculum current has contributed to its long-standing appeal among the PCPs. In the earlier cycles, to ensure quality in teaching and communication of knowledge, the trainers used to meet for a day before the start of the course and deliberate on the teaching materials and methods with the national experts, making this different from the usual training of trainers.

Currently, the teaching approach consists of two-way virtual or online instruction with presentations and discussions. Each module’s session typically lasts three to four hours. Before this, the National Expert Consultation Meet is held to ensure the strength, accuracy & relevance of all instructional materials. In other words, the purpose of this meeting is to ensure that all of the CCEBDM modules and curriculum have been updated in light of the input gathered by the secretariat from national experts, faculty, participants, and observers during the previous CCEBDM batch. The curriculum review and feedback mechanism is structured as a bidirectional and iterative process. Expert inputs, including emerging evidence and practice-relevant recommendations, are incorporated during national expert meetings. In parallel, continuous feedback is systematically gathered and documented by trained observers during course delivery across the training cycle. This ongoing exchange between content experts, faculty, and participants enables timely curricular refinements, ensuring that course content remains current, contextually relevant, and aligned with evolving evidence-based practices in diabetes management.

### Strong monitoring & evaluation mechanism

The CCEBDM employs a thoughtfully structured Monitoring and Evaluation (M&E) framework to ensure both effective implementation and ongoing quality assurance. Rather than relying on static processes, CCEBDM’s system emphasizes real-time feedback and proactive identification of obstacles, supporting a cycle of continuous improvement. The program’s approach draws upon established methodologies, such as Kirkpatrick’s Evaluation Framework^1^ and self-assessment strategies, to provide a comprehensive and evidence-based assessment of its execution and outcomes. This commitment to robust evaluation underscores the program’s dedication to maintaining high standards and achieving meaningful results.

The monitoring framework utilizes a combination of digital tools and approaches, with a customized PHFI mobile application serving as a central element. The details and the utility of this tool has been discussed at length in the section discussing the innovative approaches adopted in CCEBDM. The monitoring data and structured feedback from faculty, participants, and observers are collected digitally in a streamlined manner. Importantly, the continuous real-time feedback mechanism from the mobile application allows immediate and data-driven decision-making to make the program more impactful by facilitating real-time modifications to session duration, instructional methods, and content delivery, consequently resulting in an improved learning environment and the program’s operational effectiveness.

Apart from the iterative monitoring & feedback mechanism, a two-point survey mechanism—comprising baseline and endline questionnaires—has been embedded into the course structure to methodically assess changes in knowledge, attitudes, and practices among participants after the completion of the training. The baseline and endline instruments, which are administered at the start and at the end of the are grounded in self-assessment methodologies. The instruments include multiple-choice questions encompassing the entire diabetes care continuum: screening, diagnosis, treatment, prevention, and management. Therefore, to gauge the degree and extent of knowledge enhancement and conceptual clarity gained during the program cycle, a simple comparative analysis of pre- and post-training responses provides robust quantitative evidence.

Additionally, an endline evaluation survey is also conducted at the program’s conclusion for each cohort. The tool utilised for such an assessment gathers detailed information with respect to participant feedback on course content, delivery modalities, practical relevance, and suggestions for improvement. The collective insights from this evaluation not only form the basis for any strategic adjustments made for future iterations but also serve an important role in measuring the program’s effectiveness.

## Key innovations and pioneering aspects

### Interactive learning techniques

The CCEBDM program implemented an innovative and interactive learning environment that prioritized hands-on engagement, addressing significant insufficiencies in conventional medical education. Patient case simulations and small-group discussions enabled participating physicians to acquire the confidence and skills essential for the practical application of their knowledge [27–29]. This experience-based training approach permits learners to effectively translate theoretical concepts into actionable strategies for diabetes management in clinical settings.

The course employs a modular framework, integrating case studies, interim assignments, group discussions, and online sessions via platforms like Microsoft Teams. A pre-test and post-test system, implemented through a specialized web-based platform, enables participants to evaluate their progress and reinforces essential learning objectives. This structure provides considerable flexibility, addressing participants’ enquiries and ensuring that the training aligns with the varied requirements of busy primary care physicians. The program also includes a comprehensive learning package comprising both hard and soft copies of educational materials, enhancing accessibility and providing a robust reference resource for participants.

The CCEBDM’s distinctive blend of engagement tactics and delivery methods distinguishes it from other diabetes management training programs. Over the course of multiple implementation cycles, CCEBDM’s delivery modalities have undergone significant change, exhibiting flexibility and responsiveness to stakeholder input and contextual challenges. During the pre-COVID era, cycles 1 through 5 were carried out exclusively through in-person monthly meetings. During this phase, participating PCPs had to attend monthly contact classes at pre-identified regional training centres for roughly five to six hours on Sundays over 12 months.

Face-to-face interactions were halted in accordance with physical distancing norms and public health guidelines when the COVID-19 pandemic began. As a result, for Cycles 6–8, the program switched to an entirely online format. With record-high enrolment and participation, this digital shift greatly increased program re ach and guaranteed training continuity during lockdown periods. The online mode provided significant advantages, including cost-effectiveness and scalability for facilitators and flexibility in terms of time, pace, and location for participating PCPs. These advantages allowed primary care physicians to continue their capacity-building efforts even in the face of crises.

An important strength of the CCEBDM initiative has been its robust feedback mechanism, which has consistently informed iterative modifications in program design and delivery. With the gradual easing of the pandemic and normalization of in-person activities, both stakeholder consultations and participant feedback indicated a renewed preference for blended learning approaches. The benefits of offline learning in overcoming participants’ and instructors’ temporal, financial, and geographic obstacles are also supported by existing research [29, 30]. The current consensus in medical education is that a blended learning model, which combines the accessibility and flexibility of online learning with essential, high-impact in-person sessions, offers the most potential for a comprehensive approach to CME. Consequently, the most recent program cycle has embraced a hybrid model that incorporates both online and in-person components.

The training is conducted in small groups, maintaining an optimal trainer-to-participant ratio of approximately 1:15. This small-group format enables personalized interactions, encourages active engagement, and fosters in-depth discussions of case studies and clinical scenarios [31, 32]. The CCEBDM program is known for its intense trainer-participant interaction, which not only improves learning but also opens up possibilities for natural referral relationships. Building professional networks that can be used for implementation research, including programs like quality improvement projects, is made possible by these connections. By cultivating these relationships, the program’s influence goes beyond individual instruction and supports system-level enhancements as well as more general developments in the provision of diabetes care.

### Adaptation to remote training- building of dedicated app & website

The arrival of the COVID-19 pandemic and the subsequent shift toward virtual training modalities across public health education programs highlighted a critical need for a centralized digital structure to manage and streamline training operations. In this regard, the creation of a specially designed digital application was necessary to support both academic delivery and administrative oversight for the CCEBDM training initiative.

The application was conceived as an integrated digital platform comprising two key components: a frontend interface and a backend administrative console. In order to give participants and faculty members organized access to training materials, session schedules, assignment submissions, online tests, and feedback systems, the frontend module was created. Concurrently, the backend console was created so that facilitators and program coordinators could have centralized control over training operations. This includes the management of faculty and training centre directories, participant records, session monitoring, module dissemination, examination scoring, baseline and endline evaluations, feedback collection, and automated certification processes.

The CCEBDM program has gained several strategic advantages as a result of the implementation of this digital platform. It has improved user engagement through easier access to learning materials and tests, and it has increased operational efficiency by facilitating real-time data collection and monitoring. Additionally, it has facilitated the program’s scalability, enabling smooth expansion to meet the needs of an expanding participant base in a variety of geographical locations. Additionally, the platform facilitates reporting and communication among stakeholders and strengthens data quality, guaranteeing consistency and integrity in monitoring and evaluation outputs.

## Results

### Assessment of programme reach and perceived influence

**Fig. 1:**
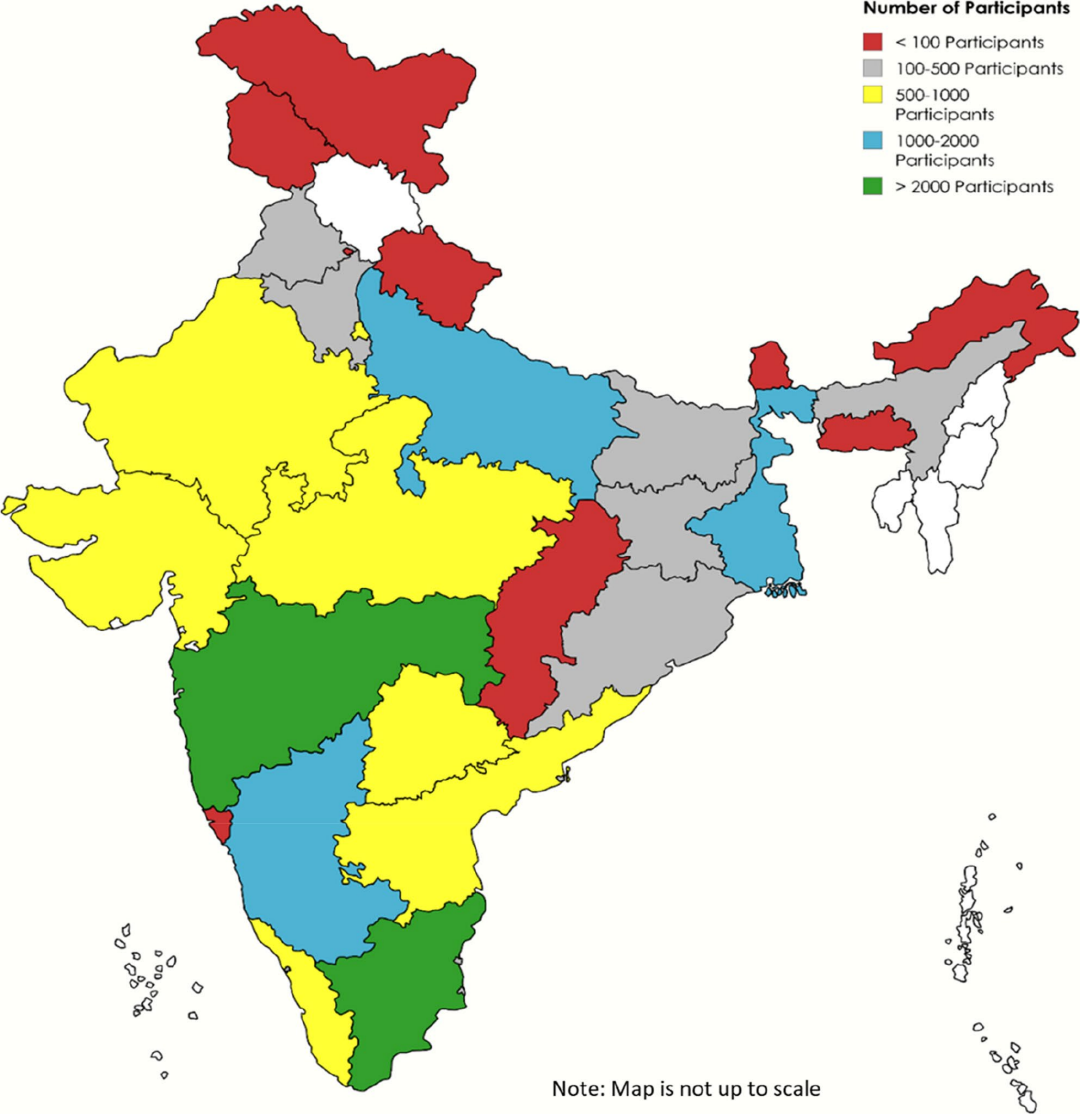
State-wise Distribution of CCEBDM Participants (*N* = 17,557)

The CCEBDM Program has trained numerous primary care doctors in various regions of India. Up to and including the present (ninth) cycle of the Program, the CCEBDM has been operational for a decade, with 104 sites across 60 districts in the State and Union Territories. The participants, representing 490 districts nationwide, demonstrate the wide coverage of the CCEBDM and its approaches to unify diabetes management protocols across the country. Figure 1 presents the distribution of participants by state for all 9 cycles of the program.

**Fig. 2:**
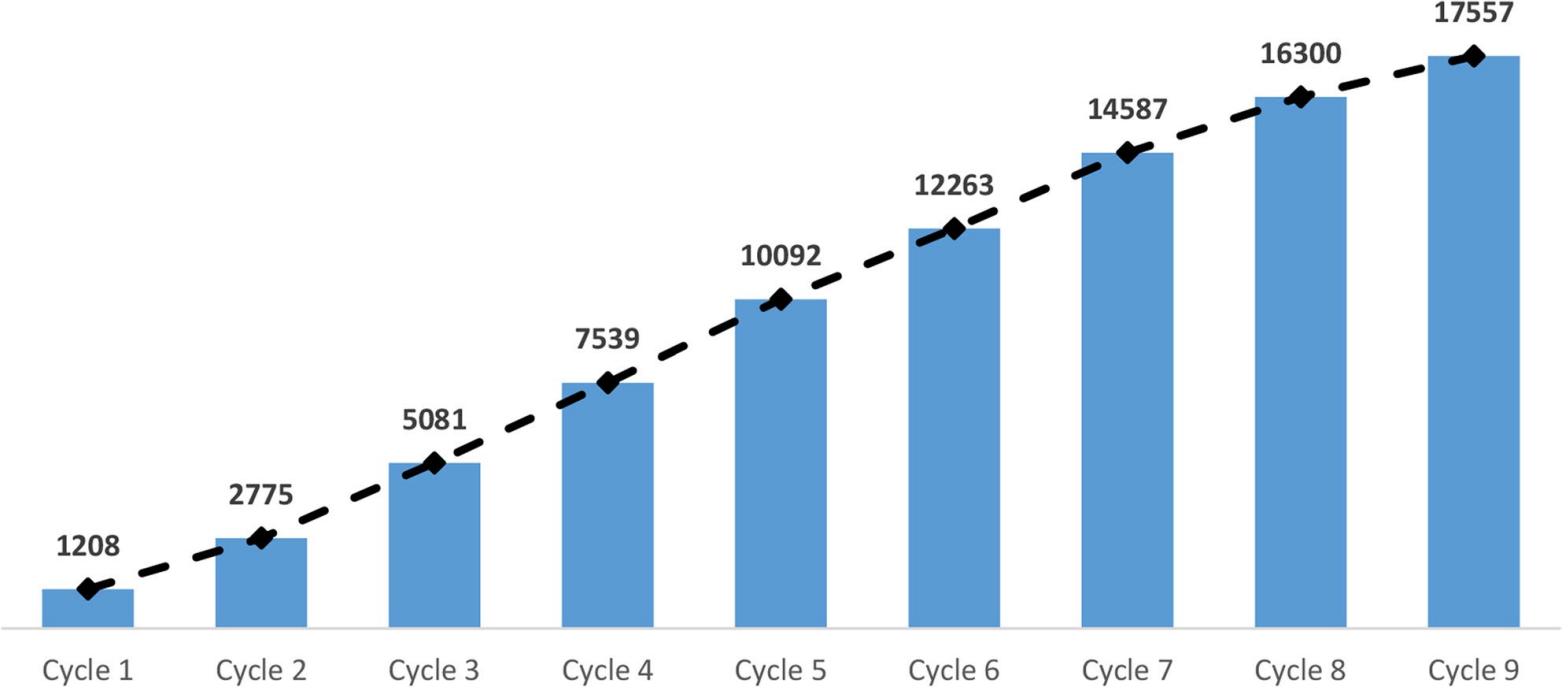
Cumulative Enrolment in CCEBDM Program, 2010–2025

To date, a total of 17,557 PCPs have enrolled for the training (Fig. 2). Notably, in earlier cycles, around 68% of participants were self-nominated, and more than one-third were nominated by public agencies, including state governments, municipal bodies, and public sector units. Recognizing its value, the governments of Kerala and West Bengal had adopted the program in 2014 to train medical officers. In the latter cycles, the entire cohort of trained participants was self-nominated. Overall, the program has trained more than 1200 primary care physicians in each cycle.

The participants comprised a diverse group of physicians, encompassing clinical experience ranging from 3 to 54 years (mean: 10.4 years), with around 40% of participants holding postgraduate qualifications. These demographics highlight how widely accepted and in-demand the program is across all professional levels.

With a strong network of 15 national experts, more than 100 faculty facilitators, and several observers to ensure quality, the CCEBDM program has expanded since 2010 to become a nationwide initiative that spans 24 States/UTs and up to 137 centres. Participant enrolment increased from 1,208 in Cycle I to 2,553 in Cycle VI. To evaluate the perceived effects of the training received in CCEBDM, a retrospective descriptive study explored participants’ self-reported perceptions of the influence of the CCEBDM training on clinical practice. The study aimed to document perceived programme influence rather than to establish causal impact.

The study included alumni from eight completed CCEBDM programme cycles. A non-probability, convenience-based purposive sampling strategy was employed. The survey was disseminated to 200 programme alumni, selected based on logistical feasibility and prior programme experience, indicating higher response likelihood among participants engaged in ongoing communications. Participation was voluntary and uncompensated. The socio-demographic characteristics of the respondents are summarized in Table 2. As such, the sample reflected self-selected respondents rather than a randomly drawn subset of all trained participants.

**Table 2:** Socio-demographic characteristics of survey respondents (*n* = 85)

Socio-Demographic Characteristics	Frequency (%)	*n*
**Gender**		
Female	20.9	18
Male	79.1	67
**Age**		
30–45	60	51
45–60	25.8	22
60 & above	14.1	12
**Practice Location**		
Urban	28.6	25
Rural	71.4	60
**Sector**		
Government	36	30
Private	59.3	51
Others	4.7	4
**Years Since Course Completion**		
Less than 5	62.4	53
5–10 years	20.0	17
10 & More	17.6	15
**Years of Experience**		
Less than 5	27.9	24
5–10	36	31
10–15	30.2	26
15 & More	5.1	5

Data were collected using an online questionnaire administered over one month, informed by time and resource constraints and declining response rates observed with extended survey duration. Eighty-five responses were received, corresponding to a response rate of 42.5%. Given the diversity of respondents in terms of clinical experience, place of practice (government, private, NGO, charitable, academic), and geographic spread, the responses were considered representative and valid for drawing meaningful inferences.

The questionnaire was developed to capture retrospective, self-reported changes in clinical practices and service delivery following programme participation. The instrument was mainly derived from the endline questionnaire of the last completed cycle, as the content is already tested and validated by the national programme experts and observers, as well as an Institutional Review Board. Additionally, the final questionnaire was internally reviewed for content relevance and clarity; however, a formal psychometric validation was not conducted.

The questionnaire captured participant demographics and professional background, perceived impact of the course on clinical confidence and practice, patient outcomes including diagnosis, management, and referrals, utilization of course resources, patient care indicators (average diabetes patients seen per month before and after training, and patients retained in follow-up care), and post-course professional development activities. The results from the survey are presented in Table 4.

#### Results of retrospective assessment survey

Respondents reported measurable improvements in both clinical practice and patient care following CCEBDM training. Before the course, participants reported an average of 80 diabetes patients per month, which increased to 139 patients per month post-training. This shows a 76% (90% C.I: 73.67 to 78.55) increase in the number of patients managed after receiving the training in CCEBDM. Given the reported increase in the average number of patients seen and the significant number of PCPs trained by CCEBDM indicate a significant impact on evidence-based management of diabetes in India through the program.

Of the 85 respondents included in the retrospective survey, 78 (91.7%) reported an increase in the number of patients seen following completion of the CCEBDM programme. In contrast, 3 participants (3.2%) reported no change, while 4 (5.1%) indicated that they were unsure or unable to recall any change. When stratified by time since course completion, reports of increased patient numbers were observed across different durations post-training, without a consistent temporal pattern. A bivariate analysis examining reported increases in patient numbers by years since course completion (≤ 5 years, 5–10 years, 10–15 & >15 years) is presented in Table 3, providing additional context on the timing of these perceived changes. Furthermore, the participants report that approximately 75% of these patients were retained for regular follow-up after training. However, the survey did not seek to identify a specific post-course time point at which changes were observed, recognizing variability in practice contexts and recall limitations inherent in retrospective assessments.

**Table 3:** Reported increase in number of patients by years of experience (*n* = 85)

Years since CCEBDM	Reported increase in patients (%)	(*n*)
Less than 5	77.73	14
5–10	75.19	33
10 & above	77.35	38
Grand Total	76.11	85

Apart from the increase in case load, participants also feel that their confidence in applying evidence-based guidelines have improved substantially, with the majority (75%) agreeing or strongly agreeing that they were more confident in managing diabetes while adhering to the guidelines. Reported improvements were highest in diagnosis (73%) and management (72.6%) of patients (Table 4).

**Table 4:** Aggregate findings from survey respondents (*n* = 85)

Self-Rated Score	Confidence in applying protocols and clinical guidelines	Change in clinical practice	Better diagnosis	Better management	Timely referrals	Overall rating of the course
1	1.1%	5.9%	1.33%	1.34%	1.34%	1.03%
2	0.5%	0.0%	0.0%	0.0%	0.0%	0.0%
3	4.0%	3.5%	2.39%	5.65%	3.22%	3.09%
4	19.0%	32.9%	23.34%	20.43%	25.74%	13.40%
5	75.4%	57.6%	72.94%	72.58%	69.71%	82.47%

**Fig. 3:**
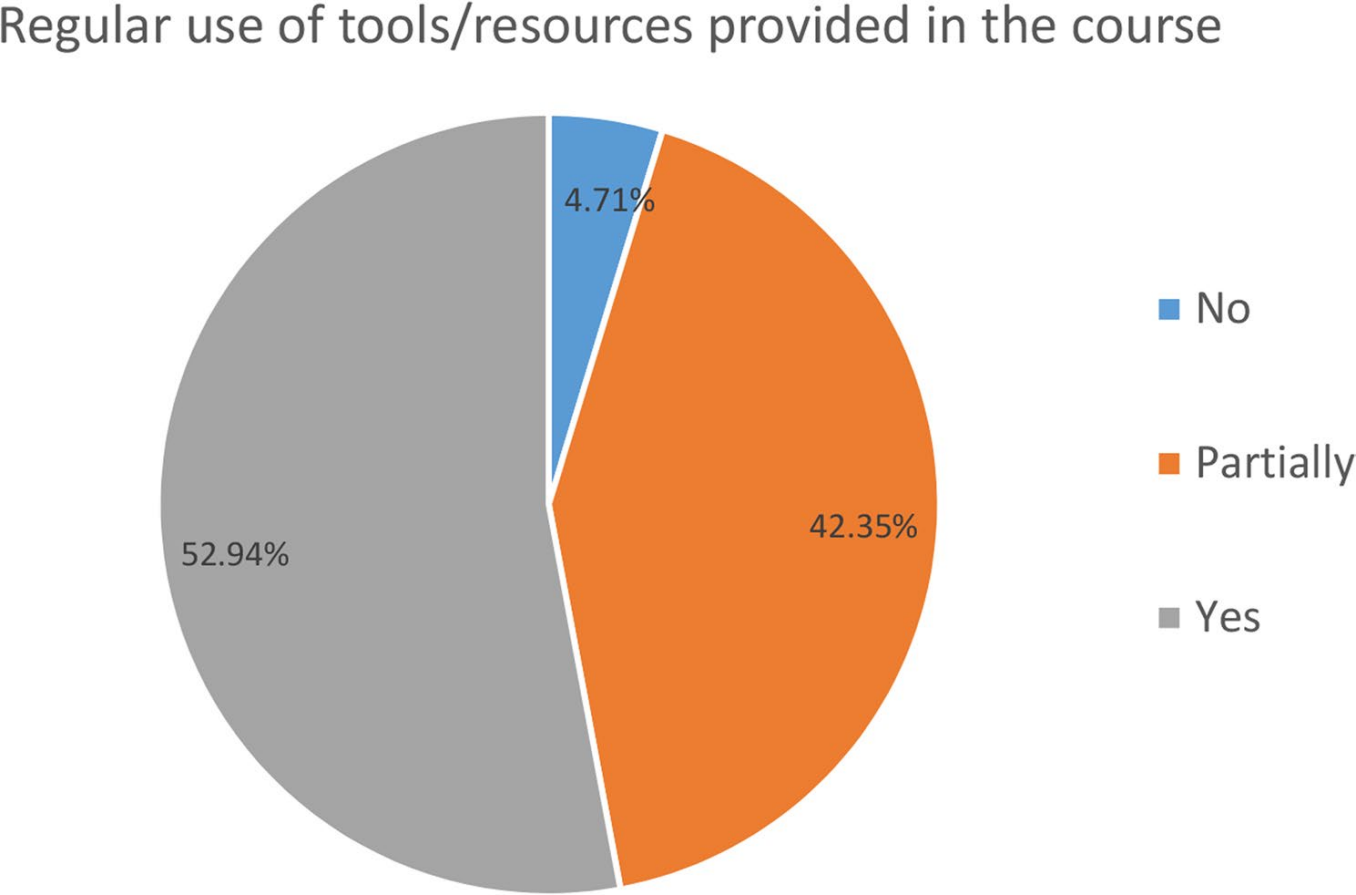
Distribution of respondents who regularly use the tools/resources provided in CCEBDM

One of the pioneering aspects of CCEBDM is that the course’s content and learning materials are of the highest standards and are regularly updated in accordance with the latest protocols and guidelines. The survey findings indicated that nearly 53% of participants concurred that the learning materials provided were pertinent to their practice and were frequently referenced post-course completion (Fig. 3). The robust process and validation involved in developing standardised learning material and their role in sustained efficacy of CCEBDM have been discussed in detail in the previous sections of the paper.

The results of this retrospective survey demonstrated that the participants of the CCEBDM program perceived that the training received had significantly improved the clinical practices of primary care providers and expanded access to diabetes care services. Respondents indicated an increase in the number of diabetes patients treated monthly following training, along with high retention rates of these patients in long-term care. Advancements in diagnosis, management, and referrals underscored the application of course content to yield concrete benefits in patient care.

#### Limitations of the study

This study has several limitations that warrant consideration when interpreting its findings. First, as a descriptive program narrative rather than a formal evaluation, it relies primarily on self-reported perceptions from a small convenience sample of 85 respondents (out of 17,557 trained participants), which may introduce recall bias and limit generalizability to the broader alumni population. The moderate response rate of 42.5% further raises the possibility of non-response bias, as respondents may differ from non-respondents in ways that could skew results, such as higher engagement with the program. Additionally, the retrospective survey questionnaire, while reviewed internally for relevance and derived from the existing programmatic evaluation, lacked specific psychometric validation or pilot testing, potentially affecting the reliability of the data collected.

Second, quantitative outcomes—such as reported increases in patient load, improved consultations, and practice changes—are based solely on participant perceptions without objective measures or causal inference, and no consolidated pre- and post-test analysis was performed due to the program’s evolving module structure and clinical guidelines over 15 years, which preclude valid aggregation across cycles. These factors underscore the indicative rather than definitive nature of the impact-related claims. Despite these constraints, the manuscript highlights the program’s sustained reach and adaptability, providing valuable insights into capacity-building efforts for PCPs in resource-limited settings. Future research with larger, representative samples and standardized assessments could address these gaps and strengthen evidence on long-term outcomes.

## Discussion- challenges for the program

A comparative analysis of the CCEBDM and other prominent medical education initiatives—such as the International Diabetes Federation’s (IDF) online course for primary care physicians, the Royal College of General Practitioners (RCGP) Diabetes eLearning platform from the UK, and the National Diabetes Education Program (NDEP) in the USA (now under the CDC)—reveals both their shared objectives and the distinctive strengths of CCEBDM. While all these programs aim to enhance the diabetes care competencies of non-specialist healthcare providers, CCEBDM stands out in its direct engagement with the specific challenges present within the Indian healthcare system.

All these educational efforts emphasize evidence-based, primary care-driven diabetes management. Yet, CCEBDM distinguishes itself through its structured, cohort-based approach, specifically tailored for India’s diverse healthcare landscape. Its evolution from in-person instruction to online and now hybrid formats demonstrates responsiveness to both pandemic-driven disruptions and learner preferences. Unlike globally oriented, self-paced modules such as the IDF’s, CCEBDM incorporates regular academic contact, ongoing mentorship, and structured feedback, thereby fostering peer learning and a substantive community of practice among Indian primary care physicians.

The program’s expansive reach across India, its formal recognition by national authorities, and integration into government-mandated training initiatives highlight its practical relevance and tangible impact. Additionally, the program’s robust digital infrastructure supports ongoing monitoring, evaluation, and academic record-keeping, components often lacking in more generic, global e-learning platforms.

Altogether, this underscores the urgent need for educational models that are both regionally relevant and practice-oriented, such as CCEBDM. As the burden of chronic diseases like diabetes grows worldwide, scalable and context-sensitive education programs, grounded in the realities of local health systems, remain essential for strengthening clinical capacity and achieving sustained improvements in population health outcomes.

## Conclusion

Programs such as CCEBDM play a substantial role in advancing global health objectives. For example, the United Nations’ Sustainable Development Goals, call for a 30% reduction in premature mortality from non-communicable diseases by 2030—a target that demands robust primary healthcare systems and well-trained PCPs. CCEBDM stands out as a model for building this capacity, particularly in diabetes care, and its structure suggests potential adaptability for managing other chronic conditions.

Over the past 15 years, CCEBDM has established a strong foundation in evidence-based diabetes care training for PCPs. it has received recognition from several international bodies, and its course model has appeared in reputable publications such as The Lancet Diabetes & Endocrinology.

Adoption has been widespread. Several Indian state governments, including Kerala, Mizoram, Madhya Pradesh, Haryana, Odisha, and Manipur, as well as the Kolkata Municipal Corporation, have implemented the program with their training programs. Major organizations like Reliance Industries have also participated. International uptake includes countries such as Myanmar, Bangladesh, Afghanistan, Rwanda, and Sri Lanka, as well as institutions like the Kabul University of Medical Sciences and the Ministry of Health of Rwanda.

The evolution of CCEBDM over 15 years illustrates its success as a pioneering initiative in diabetes education for PCPs. Its ongoing adaptation and expansion ensure continued relevance and effectiveness, positioning it as a benchmark for capacity-building efforts in chronic disease management. Lessons drawn from CCEBDM’s development and implementation could be particularly instructive for other low- and middle-income countries grappling with increasing chronic disease burdens and shortages of trained physicians.

As India’s diabetes prevalence continues to rise, programs like CCEBDM remain vital for equipping PCPs with the necessary skills to address this challenge. Sustained support and investment from policymakers, healthcare institutions, and academic bodies will be crucial for maintaining and scaling such initiatives. With ongoing commitment, CCEBDM and similar programs are poised to make a significant contribution to national healthcare improvement and to serve as models for chronic disease management in comparable settings worldwide.

## Supplementary Information

Supplementary Material 1.

## Data Availability

The datasets generated and/or analysed during the current study are not publicly available due to the data privacy laws of PHFI, but are available from the corresponding author on reasonable request.

## Abbreviations

ICMR – INDIAB: Indian Council of Medical Research-India Diabetes
NCD: Non-communicable Diseases
CCEBDM: Certificate Course on Evidence-Based Diabetes Management
DMDEA: Dr. Mohan’s Diabetes Education Academy
DMDSC: Dr. Mohan’s Diabetes Specialities Centre
PHFI: Public Health Foundation of India
PCP: Primary Care Physicians
MBBS: Bachelor of Medicine and Bachelor of Surgery
MCQ: Multiple-Choice Question
COVID-19: Coronavirus disease 2019
M&E: Monitoring and Evaluation
IDF: International Diabetes Federation
RCGP: Royal College of General Practitioners
NDEP: National Diabetes Education Program
CDC: Centre for Disease Control and Prevention

## Footnotes

1 Rouse, D. N. (2011). Employing Kirkpatrick’s evaluation framework to determine the effectiveness of health information management courses and programs. *Perspectives in health information management*, *8*(Spring), 1c.
