# Supplementary material for: Pioneering and sustaining capacity building in diabetes care: journey of the CCEBDM program
Source: BMC Med Educ. 2026 Feb 27;26:556. doi: 10.1186/s12909-026-08896-3 (PMC13049809; doi:10.1186/s12909-026-08896-3)
Supplement: Supplementary file 1 — Supplementary Material 1. [file 12909_2026_8896_MOESM1_ESM.pdf]

Impact & effectiveness survey of the Certificate Course in Evidence-Based Diabetes Management (CCEBDM)

Purpose: Participation in this survey is voluntary. The purpose of this survey is to assess the long-term impact of the Certificate Course in Evidence-Based Diabetes Management (CCEBDM) on participants' knowledge, practices, and professional development. Your responses will remain confidential and will be used only for aggregated analysis and research purposes. No personal or identifiable information will be collected or shared, and you may choose to skip any question or withdraw at any time without consequence.

Participant Details

Gender (Male / Female)	
Age	
Highest Qualification	
Years of Experience	
Sector of Employment (Govt / Pvt / NGO / Charitable/Academic)	
Place of Practice (District, State)	
Year of completing CCEBDM	
Mode of Learning (Offline/Online)	

Course Impact Assessment

Rate the following statements/questions based on your experience of completing the CCEBDM course.

(Scale for Questions 1–4: 1 = Strongly Disagree | 2 = Disagree | 3 = Neutral | 4 = Agree | 5 = Strongly Agree)

1. The content of the course were directly relevant to your clinical practice.

☐ 1 ☐ 2 ☐ 3 ☐ 4 ☐ 5

2. You are more confident in applying protocols and clinical guidelines for managing diabetes.

☐ 1 ☐ 2 ☐ 3 ☐ 4 ☐ 5

3. There are noticeable and measurable change into your clinical practice.

☐ 1 ☐ 2 ☐ 3 ☐ 4 ☐ 5

4. Patient outcomes have shown significant changes since applying course learnings.

**S.no**	**Patient Outcomes**	**Rating Scale**				
		**1**	**2**	**3**	**4**	**5**
**1.**	Better diagnosis of diabetes patients					
**2.**	Better management of diabetes patients					
**3.**	Timely referral of diabetes patients to the concerned specialist					

5. Do you regularly use tools/resources provided in the course (e.g., algorithms, case studies)? (Yes / Partially / No)

☐ Yes ☐ Partially ☐ No

6. Fill the approximate figures in the boxes given below:

(6.1) Approximate number of patients with diabetes seen in a month before attending the course ☐

(6.2) Approximate number of patients with diabetes seen in a month after attending the course ☐

(6.3) Of these, approximately how many are returning for regular follow-ups or long-term diabetes care? ☐

7. After completing the course, how have you built upon this training? (e.g., mentor others, apply digital tools, pursue more training, build a network with other clinicians)

____

____

____

8. How would you rate this course on a scale of 5? (Range 1: Needs Improvement, 3: Satisfactory, 5: Excellent)

☐ 1 ☐ 2 ☐ 3 ☐ 4 ☐ 5
